# Evolution of Pore Volume During Stripping of Lithium Metal in Solid‐State Batteries Observed with Operando Dilatometry

**DOI:** 10.1002/smll.202505053

**Published:** 2025-06-25

**Authors:** Thomas A. Schall, Till Fuchs, Janis K. Eckhardt, Tim Klunz, Boris Mogwitz, Joachim Sann, Klaus Peppler, Jürgen Janek

**Affiliations:** ^1^ Institute of Physical Chemistry Justus‐Liebig‐University Giessen Heinrich‐Buff‐Ring 17 D‐35392 Giessen Germany; ^2^ Center for Materials Research (ZfM) Justus‐Liebig‐University Giessen Heinrich‐Buff‐Ring 16 D‐35392 Giessen Germany

**Keywords:** dilatometry, lithium metal anode, pore formation, solid‐state battery, vacancy injection ratio

## Abstract

Lithium metal solid‐state batteries are prone to pore formation at the interface between the metal anode and the solid electrolyte during discharge, when the applied current density exceeds the lithium vacancy diffusion rate in the metal. This leads to contact loss and eventually battery failure. To better understand the mechanisms of pore formation at the anode interface, operando information on the total pore volume is essential. In this study, a dedicated dilatometric measurement setup for operando tracking of pore volume is presented and validated. The working principle of this method is demonstrated in a case study on symmetric lithium‐garnet cells. The combination of the dilatometric data with galvanostatic electrochemical impedance spectroscopy (GEIS) allows for a systematic study of pore formation in any metal solid‐state battery. To quantify this process, the vacancy injection ratio is introduced, as a measure of the fraction of lithium atom sites that remain empty during stripping. These experimental results reveal that, under the conditions applied, pore formation begins immediately upon stripping, even before becoming detectable by GEIS. Furthermore, it is shown that pores initially grow laterally near the surface before deepening at later stages. Based on these findings, a pore formation and growth mechanism is proposed.

## Graphical abstract

A dedicated dilatometric measurement setup for operando tracking of the pore volume is developed and validated. Using this approach, pore formation kinetics is analyzed, and the pore volume is quantified. By combining dilatometric data with impedance measurements, a deeper understanding of the pore formation mechanism is achieved, revealing that pores already begin to form at the onset of stripping. Based on these findings, a pore formation and growth mechanism is proposed.

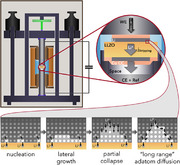

## 1 Introduction

Solid‐state batteries (SSBs) are emerging as a promising technology for next‐generation energy storage, primarily due to their potential to utilize a lithium metal anode (LMA).^[^ ^1^, ^2^, ^3^ ^]^ This capability can significantly increase the energy density compared to conventional lithium‐ion batteries.^[^ ^4^, ^5^ ^]^ However, the practical application of lithium metal solid‐state batteries is currently limited by critical interface instabilities. One of the most detrimental phenomena is contact loss due to pore formation at the interface between the solid electrolyte (SE) and the LMA during lithium stripping, limiting the available capacity and discharge current. Recent studies have further emphasized the need for a quantitative, time‐resolved understanding of pore formation by highlighting the critical role of interfacial void dynamics in performance, the onset of dendrite penetration, and mechanical failure.^[^ ^6^, ^7^ ^]^

Contact loss and pore formation occur during the discharge when lithium (Li) is oxidized at the Li|SE interface and migrates through the SE.^[^ ^4^, ^8^ ^]^ This ionic flux induces metal vacancies at the Li|SE interface. These vacancies must be replenished by diffusion within the lithium metal to preserve the interfacial integrity. If the local current density exceeds the vacancy diffusion limit in the metal, vacancies accumulate at the interface, leading to the formation of pores. Pore formation is additionally enhanced by adatom diffusion along the pore surface, as it typically proceeds faster than lithium vacancy diffusion in the bulk.^[^ ^4^, ^8^, ^9^, ^10^, ^11^, ^12^, ^13^, ^14^ ^]^

The formation of interfacial pores reduces the contact area and increases the interface resistance. As only a fraction of the contact is maintained, the current is constricted in these areas, leading to a higher local current density, which promotes further morphological instability during stripping, dendrite growth during plating, and eventually battery failure.^[^ ^9^, ^11^, ^15^, ^16^, ^17^ ^]^ While high external pressure or increased temperature can mitigate pore formation by inducing plastic deformation of the lithium metal and by increasing vacancy diffusivity,^[^ ^8^, ^17^, ^18^, ^19^, ^20^, ^21^, ^22^ ^]^ this is limited by practical concerns. High temperatures reduce efficiency and impact safety and longevity. Applying and maintaining high stack pressures during operation requires a heavy casing, which reduces the benefit of high energy density.^[^ ^16^ ^]^ Similarly, mitigation strategies such as alloying or the use of interlayers have shown potential,^[^ ^21^, ^22^, ^23^, ^24^, ^25^, ^26^ ^]^ but often come at the cost of reduced specific capacity.^[^ ^23^, ^24^, ^27^ ^]^ A mechanistic, time‐resolved insight into how pores nucleate and evolve at the Li|SE interface is therefore essential to enable the rational design of more effective and durable approaches.

To gain a deeper understanding of pore nucleation and growth, both *post mortem* and *operando* characterization techniques have been employed. *Post* *mortem* methods such as (focused ion beam) electron microscopy and electron backscatter diffraction, offer detailed morphological information on pore structures after cycling, but are limited to static, localized snapshots and cannot capture dynamic processes.^[^ ^8^, ^9^, ^15^, ^18^ ^]^ *Operando* imaging techniques, such as (synchrotron) X‐ray tomography, have enabled time‐resolved visualization of structural changes during cycling.^[^ ^28^, ^29^, ^30^ ^]^ However, their temporal and spatial resolution is insufficient to capture the early nucleation of nanometer‐scale pores. Complementary electrochemical approaches, such as galvanostatic electrochemical impedance spectroscopy (GEIS) combined with modeling, offer valuable insights into the evolution of interfacial resistance caused by pore formation and current constriction effects.^[^ ^9^, ^13^, ^19^, ^31^, ^32^, ^33^, ^34^, ^35^ ^]^ Yet, GEIS suffers from limited sensitivity to early‐stage morphological changes, as resistance and capacitance variations at the onset of void formation are often too small to detect.^[^ ^33^ ^]^

To complement the available methods with *operando* data on pore volume evolution, high‐precision measurement of the stripping electrode's height change is required. Dilatometry is a long‐established technique for detecting dimensional changes in materials with high accuracy and is typically used to study thermal expansion.^[^ ^36^, ^37^ ^]^ It has already been used for materials research in the 1960s in the Simmons‐Balluffi experiment to investigate the vacancy concentration in metals^[^ ^38^, ^39^, ^40^, ^41^ ^]^ and it remains in use today.^[^ ^42^ ^]^ Electrochemical dilatometry (ECD), introduced in the 1970s, has seen increasing use in battery development,^[^ ^43^ ^]^ though mainly for cells with liquid electrolytes.^[^ ^43^, ^44^ ^]^ In the context of solid‐state batteries, ECD has mostly been applied to study solid electrolyte (SE) processing, such as the sintering of garnet‐type SEs^[^ ^45^, ^46^, ^47^, ^48^ ^]^ or to analyze macroscopic pressure and volume changes during cycling.^[^ ^49^ ^]^ *Operando* ECD studies on lithium metal anodes in SSBs remain largely unexplored due to significant experimental challenges: the method is highly sensitive to pressure and temperature fluctuations, and nanometer‐scale resolution is required to detect the subtle changes associated with pore formation. To our knowledge, no *operando* ECD studies with the necessary precision have been reported for alkali metal‐based SSBs.^[^ ^43^ ^]^

In our work, we present a high‐precision dilatometric setup to measure the height change of the anode during stripping and to evaluate the pore volume in real‐time. The first dilatometric approach to monitor pore growth in silver anodes in SSBs was reported by Majoni and Janek in 1998 and was taken as a starting point for the current approach.^[^ ^10^ ^]^

Our study is structured in two parts: First, we introduce the methodology and its performance, including its nanometer‐scale resolution. We calculate the formed pore volume from the height change information and introduce the quantitative concept of the “vacancy injection ratio” as a measure of the lithium metal sites that remain empty during stripping.

Second, we validate our in‐house dilatometric measurement setup (see Experimental Section), demonstrate its nanometer accuracy, and apply it to study pore evolution in symmetric Li|Li_6.25_Al_0.25_La_3_Zr_2_O_12_|Li transference cells (Li|LLZO|Li) cells at constant pressure (0.07 MPa) and 25  °C under two current densities. We observe pore formation experimentally from the beginning of stripping. We demonstrate that the formed pore volume is independent of current density in the early stages. By combining dilatometry with GEIS we further show that pores grow laterally at the beginning of stripping and later grow in depth. Based on these findings, we propose a pore‐formation mechanism that supports the conclusions of Lu et al.^[^ ^9^ ^]^

Our study and the developed dilatometric measurement setup not only provide unique insight into the fundamental mechanisms of pore formation but also pave the way for the development of improved strategies to enhance the performance and reliability of SSBs with an LMA.

## 2 Measurement Methodology and Information Gain

In this section, we present an *operando* approach to quantify pore volume formation during stripping and introduce the concept of the vacancy injection ratio (VIR).

### 2.1 *Operando* Pore Volume Information

Pores form at the interface between the solid electrolyte and the LMA, making direct measurement of pore volume challenging. During the stripping process, lithium is removed from the anode, resulting in a reduction of its volume and, consequently, its height.

**Figure 1:**
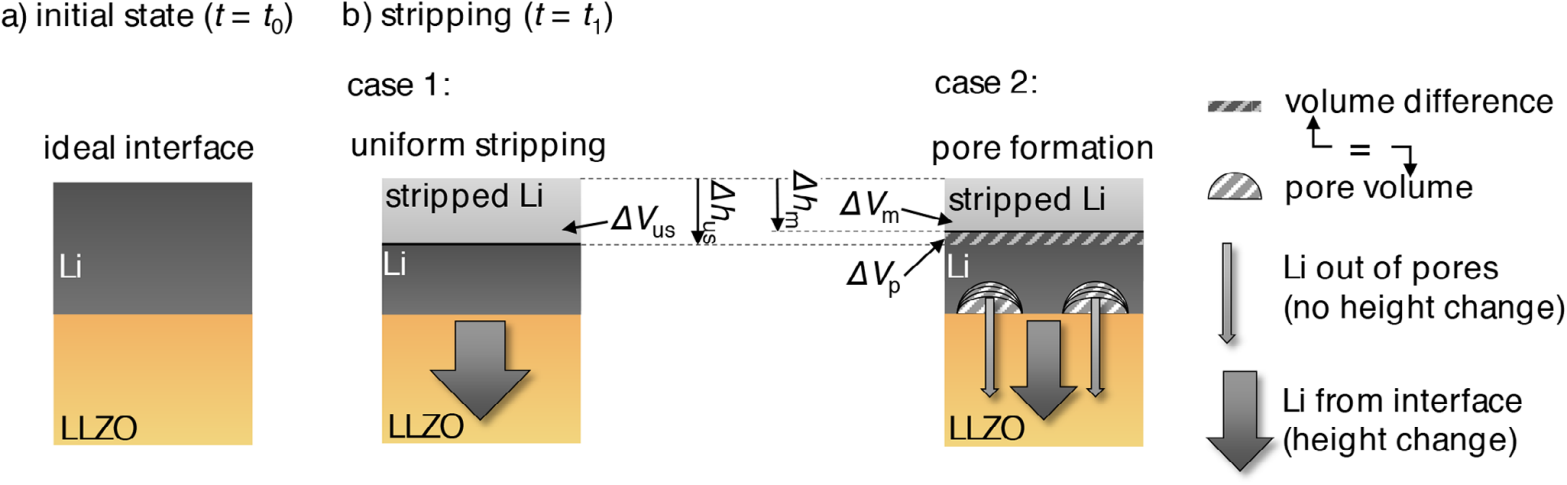
Influence of pore volume on volume reduction during stripping. a) Initial state with an ideal interface (before stripping). b) Stripping (two scenarios): In case 1 (uniform stripping), all lithium is uniformly removed from the interface, resulting in a volume reduction (Δ*V*_us_ = Δ*h*_us_ · *A*) calculated from Faraday´s law. In case 2 (pore formation), lithium is partially stripped at pore walls, leading to a lower macroscopic volume change compared to case 1 (Δ*V*_m_ = Δ*h*_m_ · *A*). The height difference between case 1 and case 2 is equivalent to the areal‐specific pore volume (Δ*v*_p_  =  Δ*h*_us_ – Δ*h*_m_).

This height change is a measurable parameter and provides a means to quantify the macroscopic volume change of the electrode. Inhomogeneous metal dissolution, resulting in pore formation on the microscale, influences the measured macroscopic volume change Δ*V*_m_. The volume change can be calculated directly from the measured height change Δ*h*_m_ when assuming a constant electrode area *A*, i.e., Δ*V*_m_  =  *A*∙Δ*h*_m_. All heights are referenced to the initial state (before stripping), where we assume that the interface contact area between the lithium and LLZO is complete (**Figure** **1**a). For the sake of simplicity, a planar interface is assumed initially. During the stripping process, two scenarios can occur (Figure 1b):

- Case 1 – *Uniform stripping*: No pores are formed because lithium self‐diffusion replenishes vacancies at the interface at a sufficient rate. Lithium is uniformly stripped, resulting in a macroscopic volume change (Δ*V*_us_) of the electrode. The volume change is equal to the volume of the stripped lithium atoms and can be calculated from Faraday´s law (see Equations S1 and S2, Supporting Information).
- Case 2 – *Pore formation*: When the applied current exceeds the rate of lithium self‐diffusion, not all vacancies are refilled, leading to pore formation on the microscale. Stripped atoms leaving enclosed vacancies at the lithium metal interface do not contribute to the measured macroscopic volume change of the electrode (Δ*V*_m_). Consequently, the reduction in electrode volume is smaller than in the uniform stripping scenario, i.e., Δ*V*_m_ < Δ*V*_us._

The difference in the volume changes between the ideal *case 1* and the nonideal *case 2* at a given time *t*_1_ corresponds to the electrochemically formed pore volume: Δ*V*_p_ *_=_* Δ*V*_us_ – Δ*V*_m_. Both hatched areas in Figure 1b represent the formed pore volume.

#### 2.1.1 Calculation of Pore Volume

The overall pore volume (Δ*V*_p_) that is electrochemically formed can be calculated from the difference between the measured volume reduction (Δ*V*_m_) and the predicted (Faradaic) volume reduction assuming uniform stripping (Δ*V*_us_), i.e., Δ*V*_p_ = Δ*V*_us_ – Δ*V*_m_. The pore volume per area (Δ*v*_p_ = Δ*V*_p_ / *A*) corresponds to the difference between the height difference measured with the dilatometer (Δ*h*_m_) and the calculated height difference for uniform stripping (Δ*h*_us_). The electrode area (*A*) refers to the anode's simplified planar geometric interface area. The area‐specific pore volume generated during the entire stripping process up to the current time (*t*) is then calculated using Equation (1):

(1) $$\Delta \;v_{p}=\frac{\Delta V_{p}}{A}=\frac{\Delta V_{\mathrm{us}}-\Delta V_{m}}{A}=\Delta h_{\mathrm{us}}-\Delta h_{m}$$

For lithium, the rate of height change for uniform stripping is Δ*h*_us_ = –8.08 nm min^−1^ for a current density of 100 µA cm^−2^, and the total height change is 4.85 µm for an areal charge of 1 mAh cm^−2^ (see Equations S3 and S4, Supporting Information)

In the case of a more complex nonplanar interface, the formal analysis remains the same, even if there is already an initial pore volume. The effective contact area may exceed that of the planar geometric interface (i.e., the cross‐sectional area). To accurately calculate the expected height change from the anticipated pore volume during uniform stripping, the current densities can be referenced to the same interface area as Δ*h*_us_. Since the simplified planar geometric area is measurable and enables more straightforward calculations, we recommend consistently using this reference area for calculating current density, height differences, and pore volumes. This assumption also holds in realistic (nonideal) cases where perfect initial contact is not present—for example, due to microscale roughness or preexisting defects at the interface. As long as the same reference area is used consistently, the analysis remains valid and allows for a meaningful comparison of relative changes during cycling.

To better interpret pore volume and estimate whether it is large or small, we introduce the concept of the vacancy injection ratio (VIR, Equation 2) for further analysis. It represents the fraction of generated lithium vacancies that are not refilled. The VIR is defined as:

(2) $$\mathrm{VIR}\;\equiv \;\;\frac{\Delta V_{p}\;}{\Delta V_{\mathrm{us}}}=\frac{\Delta v_{p}\;}{\left(\frac{\Delta V_{\mathrm{us}}}{A}\right)}\;=\frac{\Delta h_{\mathrm{us}}-\Delta h_{m}\;}{\Delta h_{\mathrm{us}}}$$

As will be shown below, the VIR is a good descriptor to quantify and discuss pore formation in detail.

**Figure 2:**
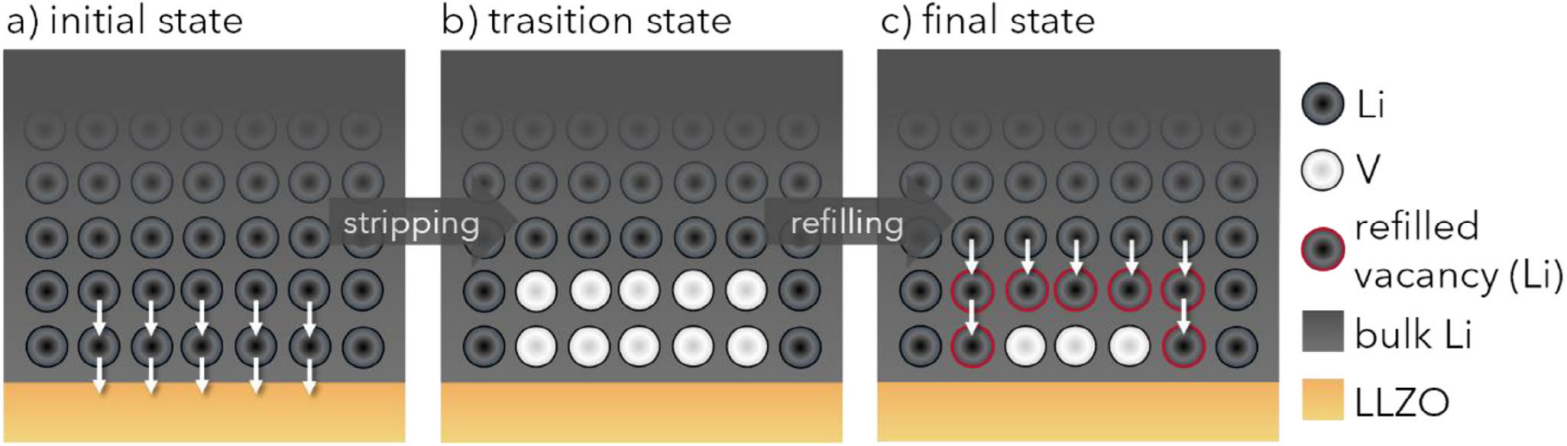
Schematic representation of a thought experiment to explain the vacancy injection ratio (VIR) with an example VIR of 0.3. Initially a), ten lithium atoms are removed by stripping, leading to ten vacancies (V) b). Seven vacancies are refilled from bulk, leaving three unfilled vacancies c). This corresponds to a VIR of 0.3.

To illustrate the VIR concept, we propose a thought experiment shown in **Figure** **2**. In this conceptual scenario, it is assumed that the process steps occur sequentially rather than simultaneously. In the initial state a) an ideal interface is assumed. In the transition state b), ten lithium atoms are removed from the Li|LLZO interface during stripping, creating ten vacancies (Figure 2b). Subsequently, seven of these vacant lithium sites are refilled through bulk lithium, leaving three vacancies unfilled in the final state (Figure 2c). This corresponds to a VIR of 0.3.

Based on this thought experiment, the VIR during stripping is 30%. This indicates that 70% of the injected vacancies are refilled, while 30% remain unfilled. Ideally, stable interfaces should show a very small VIR that remains low during continuous operation. We like to add that the VIR also enables the calculation of additional descriptors, such as the pore refilling current (see Equations S5 and S6, Supporting Information).

### 2.2 Influence of Plastic Deformation and Creep on Pore Volume Measurements

The experimental setup used for these measurements is described in the Experimental Section.

Our dilatometer operates like a classic capacitive push piston dilatometer. Height changes are measured by tracking capacitance variations, which occur when the distance between a capacitor plate connected to the cell and a fixed reference capacitor plate changes. This setup allows the application of pressure to the sample. The weight applied to the cell (and consequently the pressure) is calculated by considering all components above the measured electrode. Thus, a minimal pressure (180 g or 0.06 MPa in the present case) is always present due to the weight of the components positioned between the measured electrode and the capacitor plate.

During measurements, the constant pressure applied to the anode induces *elastic and plastic deformation*, as well as *creep*, all of which influence the recorded height change. These effects lead to an additional height reduction of the anode, resulting in a greater measured height change. Under constant pressure, the strain rate due to mechanical deformation decreases over time, leading to a progressively smaller height reduction.^[^ ^50^ ^]^ Therefore, before electrochemical measurements (*i.e*., the stripping process) are started, the mechanically induced height reduction is carefully analyzed. As expected, this reduction initially occurs at a high rate before decreasing to a low, approximately constant rate, that can be well distinguished from the electrochemically induced changes (Figure S1a,b, Supporting Information).

To ensure that mechanically induced height changes are negligible compared to electrochemically induced height changes, sufficient time is allowed before starting stripping. In this study, a height change of < | − 0.1| nm min^−1^ is measured prior to stripping, which can be attributed to creep. Since the influence of creep is more than one order of magnitude lower than the height change during stripping (> | − 5| nm min^−1^ for 100 µA cm^−2^) it is considered negligible, and no mathematical correction is applied. At higher pressures (in this study 0.07 MPa are applied) where creep rates may be higher, corrections may be necessary in future analyses.

For uniform stripping, height reduction is calculated using Faraday's law, without accounting for mechanical deformation (Equations S1–S4, Supporting Information). However, since the measured height reduction includes contributions from mechanical deformation, this may lead to a slight underestimation (< ≈2%) of the pore volume.

In general, plastic deformation and creep counteract pore formation during stripping.^[^ ^31^ ^]^ As pore formation reduces the remaining contact area, local pressure increases, enhancing mechanically induced height reduction. Importantly, this increased height reduction partially fills the pores and does not represent a measurement error. The increased height reduction can be observed in (Figure S1c, Supporting Information).

## 3 Results and Discussion

Experimentally measuring pore volume is challenging, as it requires detecting length changes in the order of just a few atomic layers per minute with high accuracy. Such measurements are only feasible under strictly isothermal and isobaric conditions with a highly sensitive setup. Additionally, to measure LMAs, the measurement cell must be both friction‐free and airtight. To meet these requirements, we have developed a custom in‐house solution, detailed in the Experimental Details section under Measurement Setup.

To ensure the accuracy and precision of our dilatometric measurement setup, we first validate it using a well‐controlled reference sample. We then demonstrate the capabilities and advantages of *operando* pore volume analysis through a case study conducted at two different current densities for lithium stripping at the Li|LLZO interface.

### 3.1 Validation of Accuracy and Function of the Dilatometer

The accuracy and functionality of the dilatometry setup were validated by integrating a piezo actuator as an active sample within the frictionless measuring cell (see Figure S2, Supporting Information). This enables precise control of the height change inside the cell, allowing predetermined displacements to be compared with measured values. The piezo actuator has a maximum displacement range of 30 µm. For validation, the actuator was driven downward from 30 to 0 µm at varying velocities ranging from 6000 to 5 nm min^−1^. Before and after each validation measurement, the actuator remained stationary for 60 min to ensure mechanical stability. Between measurements, it was repositioned upward at a constant velocity of 6000 nm min^−1^.

At displacement rates of 6000, 1000, 100, and 10 nm min^−1^, the measurement error was 0.5%. At a displacement rate of 5 nm min^−1^, the error increased to 1.1% (see Figure S3, Supporting Information). This confirms the mechanical accuracy and reliability of the dilatometry setup.

In general, temperature fluctuations can introduce measurement errors due to thermal expansion effects. Temperature stability was monitored using three thermocouples placed at the measurement device. Based on test measurements, the thermal expansion was determined to be Δ*L*/Δ*T* = 10^−6^ m K^−1^, meaning that a temperature change of 10^−2^ K would correspond to a height change of only 10 nm. During all measurements, temperature was continuously monitored to detect any potential thermal‐induced errors. In all measurements presented in this work, temperature fluctuations remained below 10^−2^ K, ensuring that thermal expansion effects were negligible.

### 3.2 *Operando* Pore Formation Measurements at the Li|LLZO interface

Having established a reliable measurement framework, we now focus on measuring pore formation *operando*. To isolate the effects of stripping, only the height change of the stripping electrode should be measured in the dilatometer. To achieve this, the plating electrode is positioned within a recess, mechanically excluding it from the measurement. As a result, any mechanical displacement caused by lithium plating is taken up within the cavity of the recess and does not contribute to the measured signal. The recess design is detailed in the Experimental Details section under Measurement Setup.

This measurement setup enables the *operando* analysis of pore formation, particularly in the early stages during nucleation. To the best of our knowledge, no previous experimental work has investigated pore formation before constriction effects become detectable. Low current densities are particularly valuable for understanding the underlying mechanisms, as they provide a prolonged phase at the beginning during which constriction effects are not detectable.^[^ ^33^ ^]^

While measuring the height change at small current densities is experimentally challenging, our setup demonstrates high accuracy even for the small height changes expected at 100 µA cm⁻^2^ for Lithium. Therefore, we examine pore formation during stripping at current densities of 100 and 200 µA cm⁻^2^. Notably, this approach can be readily extended to target current densities and metals with larger molar volume and therefore larger height change (*i.e*., sodium or potassium).

**Figure 3:**
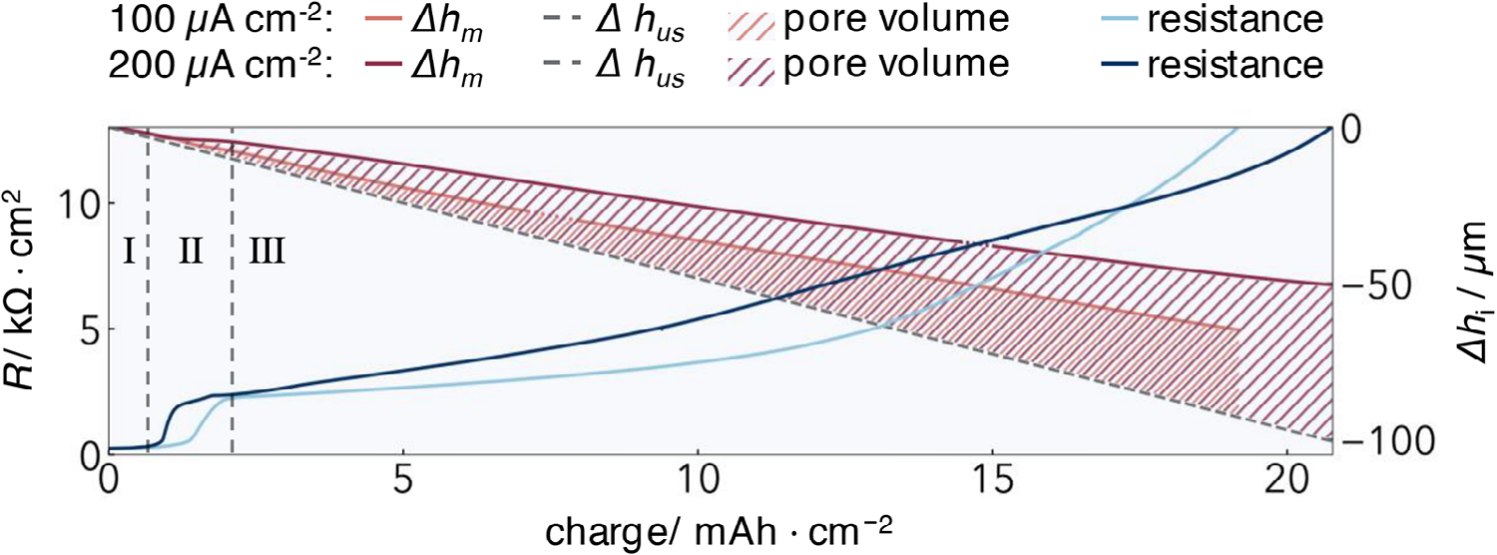
Dilatometric height change and corresponding resistance for stripping experiments conducted at 100 and 200 µA cm^−2^ at a Li_id_|LLZO|Li_id_ cell at 25 °C, under an added pressure of 0.07 MPa, until a resistance of 13 kΩ cm^2^. The dashed gray line represents the calculated height change for uniform stripping, while the resulting pore volume is indicated by hatching. In both measurements, a brief failure in data recording occurred; the missing data were interpolated and are shown as dotted lines.

Stripping experiments were performed using symmetrical cells with ideally reversible lithium electrodes on both sides (Li_id_|LLZO|Li_id_)^[^ ^8^ ^]^ at a constant pressure of 0.07 MPa. As described above, the plating electrode did not enter into the dilatometer signal. One cell was operated with a current density of 100 µA cm^−2^ (at (25.085 ± 0.005) °C) and the other with 200 µA cm^−2^ (at (25.080 ± 0.005) °C). The results of both cells were plotted up to a resistance of 13 kΩ cm^2^ in **Figure** **3**. *Post mortem* analysis was conducted by peeling off the anodes and examining them via SEM imaging (Figure S4, Supporting Information), where pore formation is clearly visible.

Figure 3 shows the height change Δ*h*_m_ (red line) and the corresponding DC resistance of the cell (blue line) versus the stripped charge (*i.e*., amount of lithium). The grey dashed line represents the height change as calculated under the assumption of uniform stripping (Δ*h*_us_  =  −4.85 µm / (mAh cm^−2^)). The difference, as marked by the hatched areas, corresponds to the formed pore volumes for both current densities (Δ*v*_p_, Equation 1).

**Figure 4:**
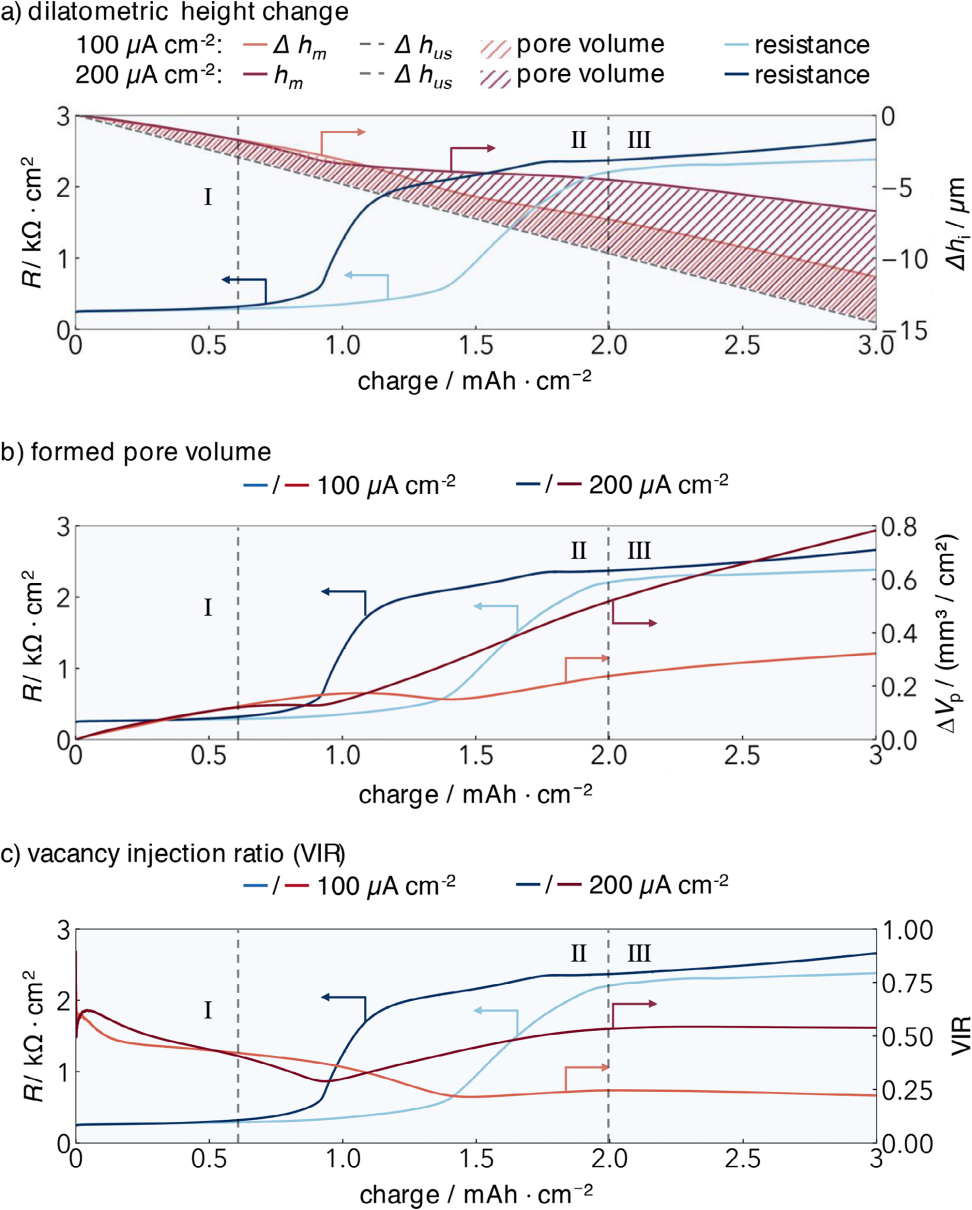
Detailed analyses of the first 3 mAh cm^−2^ of the stripping experiments form Figure 3: a) height change; b) formed pore volume; c) vacancy injection ratio (VIR).

The resistance profiles show an S‐shaped curve, suggesting three distinct regimes: Regime I at the beginning (0 – 0.6 mAh cm^−2^), Regime II during the resistance increase (0.6 – 2 mAh cm^−2^), and Regime III from the resistance increase until the cut‐off resistance (from 2 mAh cm^−2^). As low resistance operation is desired for practical applications, the following discussion will focus on the three defined regimes in the first 3 mAh cm^−2^, as shown in **Figure** **4**.

**Regime I**: The height decreases continuously from the onset of stripping. Since the measured height change Δ*h*_m_ is smaller than the calculated (Faradaic) height change Δ*h*_us_ from the beginning, this corresponds to Case 2 in Figure 1. Consequently, pore formation begins immediately upon stripping but remains undetected in the resistance signal. Classical resistance or impedance measurements – commonly used in the literature – are unable to detect early‐stage pore formation,^[^ ^33^ ^]^ as the resistive effect of a small pore area at the interface is too small.

A change in resistance is observed only after 0.6 mAh cm⁻^2^ (marking the end of Regime I), which is consistent with simulation results showing that a significant resistance change requires a certain pore area at the interface.^[^ ^33^ ^]^ This highlights a key advantage of the dilatometry method over conventional techniques, as it enables detailed tracking and analysis of pore formation from the very beginning.

During Regime I, the evolution of resistance and height change is identical for both current densities. Thus, the initially formed pore volume is independent of current density and only depends on the amount of stripped lithium (Figure 4b). This is also reflected in the VIR, which is also close for both current densities in this regime. The VIR curve is slightly stretched for 100 compared to 200 µA cm⁻^2^, meaning that the pore‐forming steps occur at the higher current density at a lower charge. Initial deviations in the VIR are attributed to measurement uncertainties, as the differences between the measured pore volume change and the calculated uniform stripping volume are small relative to the error margins.

At a capacity of 0.5 mAh cm⁻^2^, the VIR reaches 0.5 for both current densities, indicating that half of the stripped lithium is replenished, while the other half contributes to pore formation. A rough estimate gives an area‐specific pore volume, i.e., an average pore thickness of ≈2.4 µm, assuming that 50% of the contact area is homogeneously covered with pores (the resistance starts to increase at this point).^[^ ^33^ ^]^

**Regime II**: Between 0.6 and 2 mAh cm⁻^2^, the resistance rises exceeding 2 kΩ cm⁻^2^ for both current densities, occurring at a lower capacity for 200 than for 100 µA cm⁻^2^.

At the point where the resistance rises, the pore volume formed decreases to a minimum before increasing again. This minimum in formed pore volume coincides with a minimum in the VIR. This indicates that two processes are occurring simultaneously. One process reduces pore volume (*i.e*., partial collapse of the pores) and one process increases the resistance (*i.e*., reduction of the remaining contact area). This is discussed below.

The minima in pore volume and resistance after the increase are comparable for both current densities. The minimum occurs at a smaller charge for the higher current density. After reaching the minimum, the pore volume at 200 µA cm⁻^2^ increases at a higher rate than at 100 µA cm⁻^2^. Approaching 2 mAh cm⁻^2^ with 200 µA cm⁻^2^ (end of Regime II), the pore volume is approximately twice that of 100 µA cm⁻^2^. The VIR is rising from the minima to the end of Regime II at 2 mAh cm^−2^, and it is almost double for the higher current density. Clearly, the higher current density causes more vacancy injection, *i.e*., pore formation. The same amount of lithium is stripped in only half the time, and diffusion is too slow at the higher current density to refill the same fraction of vacancies. The VIR reaches an approximately constant value of 0.24 for 100 and 0.53 for 200 µA cm⁻^2^, meaning that at 100 µA cm⁻^2^, approximately every fourth vacancy contributes to pore formation, and at 200 µA cm⁻^2^ approximately every second. We note that we made a simple estimate of the current density that allows stable stripping some years ago on the basis of vacancy diffusion in lithium, resulting in ≈100 µA cm⁻^2^.^[^ ^8^ ^]^ In view of the present results, this estimate appears to be too optimistic. However, our current experiments were run at a very low applied pressure of only 0.07 MPa which is much lower than in most other experiments reported in literature.

**Regime III** (from 2 mAh cm⁻^2^) is characterized by a continuous increase in pore volume, while the VIR remains approximately constant. The resistance stabilizes at a plateau and increases only slightly at very high pore volumes (Figure S5, Supporting Information). In this regime, both the pore volume and VIR are approximately twice as high at 200 compared to 100 µA cm⁻^2^, meaning that the pore volume relative to the stripped charge increases twice as fast at 200 compared to 100 µA cm⁻^2^ and four times as fast relative to the stripping time. This clearly indicates a dependence on current density at this stage.

The analyzed stripping experiments clearly demonstrate the advantages of the dilatometric setup, providing valuable insights into pore formation. Evidence of pore formation is observed from the very beginning of the measurement (Regime I). The results indicate that, initially, pore formation is independent of current density (Regime I) but later decreases to a minimum (Regime II) before transitioning into a current density‐dependent regime (Regime III). These findings highlight the ability of the dilatometric method to capture previously inaccessible details of the early stripping phase, offering new and valuable insights into this critical stage. In the following, the results of GEIS measurements will be discussed, before we will try to interpret the combined results.

### 3.3 Morphological Analysis Using GEIS

To further expand our understanding and gain additional information, the dilatometric measurements are combined with GEIS. This approach provides a more comprehensive picture of the underlying mechanisms, as the limitations of each method are mitigated by the strengths of the other. While the dilatometer measures the total formed pore volume without distinguishing between different pore geometries, GEIS allows for a qualitative assessment of the remaining contact area and its evolution over time, when current constriction become pronounced enough for detection.^[^ ^33^ ^]^ This combined analysis enables the investigation of the simultaneous increase in resistance and decrease in pore volume in Regime II.

To combine the operando GEIS measurements (for selected spectra see Figure S6, Supporting Information) with the dilatometric data, the impedance data are transformed into the time domain. For this purpose, the distribution function of each individual spectrum *γ*(*τ*) is calculated using a distribution of relaxation times (DRT) analysis.^[^ ^51^ ^]^ To enable a direct comparison of the two methods, a contour plot is generated from the hundreds of distribution functions *γ*(*τ*). For this purpose, the temporal development of the amplitude of the distribution function at different relaxation times *τ* is color‐coded.

Each charge transport/transfer process can be assigned to a range of relaxation times.^[^ ^52^ ^]^ Bulk transport in the LLZO solid electrolyte can be assigned in the relaxation time range from 10^−7^ to 10^−6^ s, transport across grain boundaries (GB) mostly occurs in the time range from 10^−6^ to 10^−4^ s, and interface processes can be found between 10^−4^ and 10^−2^ s.^[^ ^9^, ^52^, ^53^ ^]^ The contour plot of the DRTs provides a measure of the resistance evolution of the individual transport processes. Insights into the composition of the overall resistance can be drawn from the temporal development of the processes in terms of relaxation time and resistance. In particular, the extent to which the interface morphology changes during the stripping experiment can be extracted.

**Figure 5:**
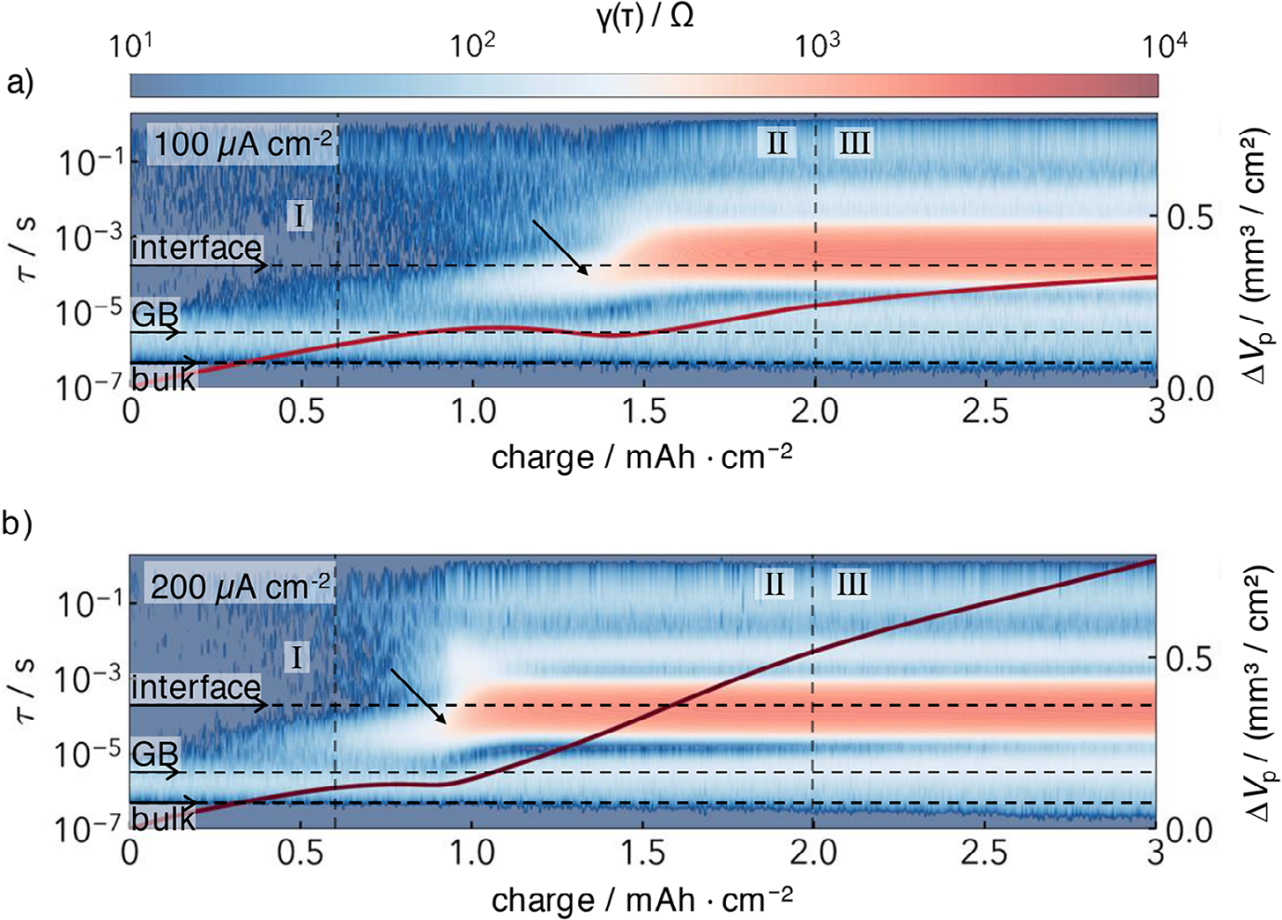
DRT contour plot combined with the total pore volume (per area) formed with time from Figure 4.

**Figure** **5** shows the DRT contour plots at different current densities overlaid with the pore volume evolution data from Figure 4b. The relaxation times *τ* are plotted along the left *y*‐axis, with the corresponding transport processes assigned.^[^ ^8^, ^54^ ^]^ This kind of data representation allows us to quickly see changes in the different transport processes together with the pore volume information.

Initially (Regime I), there are two different signals (“two bars”) in both DRT contour plots that can be assigned to bulk transport and transport across grain boundaries (GB) in the SE. These two signals remain constant with the stripping time. There is no initial interface signal.

In Regime II, an interface signal arises from ≈0.7 mAh cm⁻^2^ for 100 µA cm⁻^2^ and at ≈1 mAh cm⁻^2^ for 200 µA cm⁻^2^. It is constant in the considered range up to 3 mAh cm⁻^2^ for both current densities (Regime III). This matches the S‐shaped resistance curve in Figure 4. The interface signal occurs together with the increase in resistance and remains constant according to the plateau in the resistance. The increase in resistance can be explained by the current constriction effect.^[^ ^33^ ^]^

In Regime II the pore volume formed decreases to a minimum. The DRT contour plots reveal that the influence of the interface resistance increases at the same time. This indicates the occurrence of constriction effects at the interface. The 2D contact decrease of the interface is predominantly included in the impedance, the influence of the pore depth is here negligible.^[^ ^33^ ^]^ However, a reduction in the pore depth leads to a shift in the relaxation times, as highlighted by the arrows in Figure 5.^[^ ^33^ ^]^ This shift has previously been reported by Haslam et al. in relation to relaxation after stripping.^[^ ^53^ ^]^

While the contact area decreases, lithium is stripped from the remaining interface at higher local rates and the pore area increases. This process begins at the local maximum of the pore volume. The reduction of contact area causes a localized stress amplification. The locally higher pressure compresses the pores and thus leads to a decrease in the pore height and therefore a decrease in pore volume at the same time as the contact area decreases.

As the interfacial resistance reaches a plateau in Regime III, pore growth in depth is indicated. As the control parameter applied pressure does not scale with the stripped capacity, this indicates an influence of the pressure‐induced creep on the pore volume. The effects are quantitatively comparable for both current densities.

The combined analyses show that the pore height is reduced while the pore area is increased in Regime II. As the concept of VIR assumes a surface‐near process and the VIR is calculated as an average of the whole process up to the point in time on the *x*‐axis, it is unclear whether the assumptions for the VIR are still valid ongoing from the pore height reduction in Regime II.

Clearly, the combination of dilatometric measurements and DRT analysis provides a more comprehensive understanding of pore formation. The dilatometric data confirms that pore volume expands in all regimes. Initially, the formed pore volume is independent of current density, suggesting that small pores nucleate in this period. In this early stage, changes in the contact area cannot yet be detected by GEIS.^[^ ^33^ ^]^ It is hypothesized that these pores initially expand primarily laterally, *i.e*., in area, until a constriction signal becomes measurable (Regime II), leading to an increase in total resistance. As the interface area decreases, the local pressure rises, causing partial pore collapse by creep (between 1.1 and 1.4 mAh cm⁻^2^ for 100 µA cm⁻^2^ and between 0.7 and 0.9 mAh cm⁻^2^ for 200 µA cm⁻^2^). However, active contact is not re‐established, as evidenced by the continued increase in the constriction signal. This suggests that the reduction in pore height occurs without restoring the electrochemically active interface.

In Regime III, the total pore volume continues to increase, while the electric constriction effect—and thus the active contact area— remains approximately constant. This implies that the stripped lithium is mostly originating from regions not directly at the interface. It is transported along the pore metal surface (i.e., the pore “walls”) toward the SE interface areas, indicating a “long‐range” adatom diffusion mechanism – as already suggested in the case of silver electrodes.^[^ ^10^ ^]^ In this phase, it appears to be energetically more favorable for lithium to be transported out of the pore rather than to further reduce the remaining contact area at the interface. These findings suggest that diffusion processes evolve during pore formation, with “long‐range” adatom diffusion becoming particularly relevant in Regime III.

**Figure 6:**
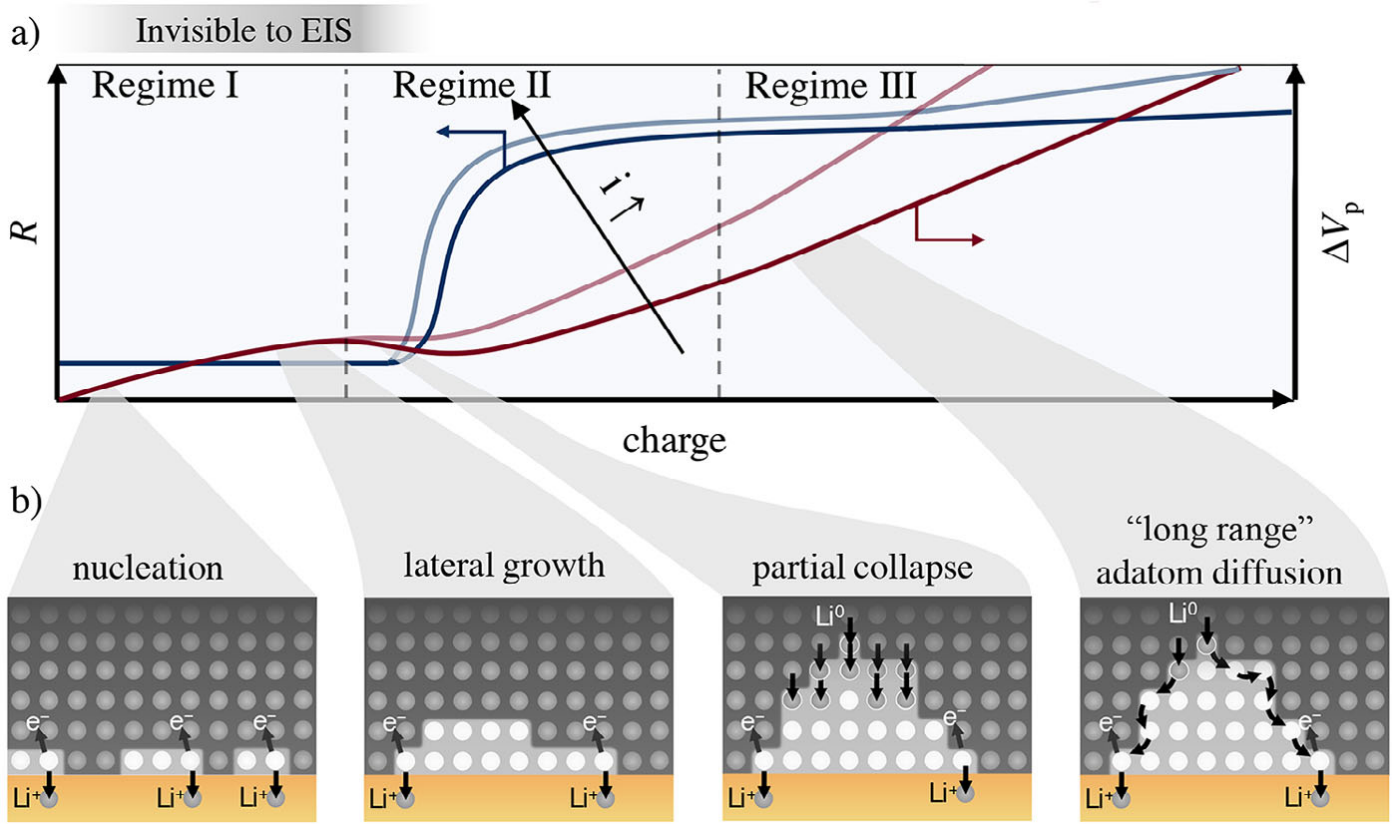
Pore formation mechanism: a) Pore volume and resistance evolution b) Atomistic view. Pore nucleation begins at the onset of stripping. Once nucleated, pores primarily expand laterally. Though lateral growth continues, partial pore collapse occurs. Subsequently, the contact area stabilizes, while lithium is transported from the top of the pores through “long‐range” adatom diffusion.

Based on the results, we propose a pore formation and growth mechanism (**Figure** **6**). At the onset of stripping pores begin to nucleate if the injected vacancies cannot be refilled effectively (Regime I). During nucleation, the formed pore volume remains independent of current density. After nucleation, pores primarily expand laterally (Regime II) at the cost of the contact area. As pore growth continues, electric constriction becomes apparent for GEIS, and partial pore collapse occurs, although lateral expansion persists. Subsequentially, the contact area and constriction stabilize while pores grow in depth (Regime III). During this phase, lithium is transported from the top of the pore via “long‐range” adatom diffusion. The deepening of pores leads to a gradual increase in resistance until failure occurs due to contact loss (not shown in Figure 6).

### 3.4 Further Discussion of Pore Formation and its Role

Lu *et al.* discussed the pore formation mechanisms at the Li|Li_7_P_3_S_11_ interface at a stack pressure of 5 MPa at 25 °C and variable current densities using impedance data, *operando* optical observations, and SEM images,^[^ ^9^ ^]^ identifying three stages. In Stage I, pores nucleate while the resistance remains low, and the pore volume remains small. This phase lasts until ≈1 mAh cm^−2^, which aligns with our observations from the dilatometric study, where the resistance remains low up to ≈1 mAh cm^−2^. During this stage, the pore volume is independent of current density, consistent with the nucleation process. As described by Lu *et al.*, the number of pore nuclei differs with the current densities, but this cannot be detected by dilatometry. Stage II is characterized by planar void growth, where individual nuclei begin to grow and coalesce into larger pores, depending on the number of nuclei. This phase occurs between ≈1 and 2 mAh cm^−2^. Stage II corresponds to the increase in resistance observed in Figure 4 as well as the rise in interfacial resistance shown in Figure 5. Planar void growth is further supported by the simultaneous increase in interfacial resistance and decrease in pore volume. In Stage III, pores grow in height. This phase is consistent with the resistance plateau observed in Figure 4, as well as the faster pore growth at higher current densities. To summarize, Stages I to III correspond in good approximation to Regimes I to III from our work and give a consistent picture of pore formation and growth.

We like to add that the S‐shaped resistance profile has previously been observed at Ag|AgI electrodes during stripping.^[^ ^10^, ^55^ ^]^ In these studies, periodic phenomena during the anodic dissolution of silver were observed. The S‐shaped voltage profile occurred at applied pressures higher than atmospheric pressure but below the threshold required for oscillations. These oscillations have been attributed to the periodic formation of pores followed by pressure‐induced collapse. When the pressure was insufficient to induce pore collapse, a plateau was reached instead.

In our study, a similar plateau is observed at a relatively low applied pressure of 0.07 MPa. During the resistance increase, we detect a drop in pore volume (at ≈1.4 mAh cm⁻^2^ for 100 µA cm⁻^2^ resp. 0.9 mAh cm⁻^2^ for 200 µA cm⁻^2^), consistent with observations made by Majoni and Janek in their dilatometric oscillation studies.^[^ ^10^, ^55^ ^]^ However, while their study shows a significant reduction in pore volume accompanied by a decrease in resistance, Figure 4 indicates only a slight decrease in pore volume, which appears insufficient to account for a resistance reduction. This suggests that the applied pressure may be too low to fully collapse the pores and trigger oscillations in our experiments. Pore formation can be detected more sensitively in the dilatometer than with EIS and therefore changes in pore formation,^[^ ^33^ ^]^ indicating oscillations can also be detected very sensitively.

## 4 Conclusion

This study presents a methodology, and an in‐house (customized) dilatometric measurement setup for *operando* analysis of pore formation in the lithium metal anodes of solid‐state batteries (SSB). By capturing nanometer‐scale height changes under isobaric and isothermal conditions, this method enables precise evaluation of electrochemically formed pore volume and the vacancy injection ratio during stripping. This adds independent and novel *operando* information to the understanding of a typically buried interface, that cannot be obtained by other techniques.

The nanometer scale precision of the setup was validated using a piezo actuator confirming its accuracy and suitability for measuring pore formation under challenging conditions, such as at low current densities. By analyzing pore formation at current densities of 100 and 200 µA cm^−2^ in symmetric Li|LLZO|Li cells, we experimentally demonstrated that pore formation begins immediately upon stripping. Furthermore, our results show that in the nucleation stage, the pore volume (relative to the stripping capacity) increases independently of current density. As stripping progresses, an S‐shaped increase in resistance is observed, marking a transition to a current density‐dependent regime. The presence of S‐shaped behavior and oscillation phenomena in silver cells suggests that similar oscillations may also occur in lithium metal solid‐state batteries. However, further experimental studies are needed to confirm this hypothesis.

By combining dilatometric measurements with GEIS the limitations of each method are mitigated by the strengths of the other. Our analysis reveals that pores initially expand laterally. Before significant pore deepening occurs, partial pore collapse is observed, coinciding with the first measurable constriction effects in GEIS. When the pores grow in depth the lithium originates from the top of the pore through a “long‐distance” adatom diffusion. Based on these findings, we propose a pore formation mechanism that describes the nucleation, lateral expansion, and deepening of pores during stripping. In particular, regime I is of high practical relevance, as the pore volume is independent of the current density and it should be attempted to extend this regime to larger capacities. Further extended and systematic work on pore formation at higher applied pressure and temperature, as well as on the role of interlayers, will be done in the next steps. Based on the VIR, the influence of pressure, temperature, and intermediate layers can be systematically analyzed.

This method opens the door to a wide range of further analyses, including pore formation at low currents, cathode breathing, alloy anode behavior, measurement of volume changes due to undesired processes such as solid electrolyte interphase (SEI) formation, and the detection of ionic conduction. In particular, it may also be extended to cumulative multi‐cycle experiments, such as critical current density (CCD) tests, to investigate long‐term effects like pore refilling and accumulation. By complementing and overcoming the limitations of existing techniques, this approach helps to gain a better understanding of further research and development in the field, paving the way for the optimization of SSB technology. By enhancing our understanding of pore dynamics, this approach contributes to the advancement of more robust and efficient energy storage solutions.

## 5 Experimental Section

To perform pore volume measurements, a setup capable of detecting height changes with nanometer‐scale accuracy, while separating the height change of a single electrode from the height change of the whole cell, was required.

### Measurement Setup—Operando Measurement of Nanometer Scale Height Changes

A suitable technique for high‐resolution measurements was capacitive dilatometry, which was based on the principle of a plate capacitor with variable plate distance. Changes in the distance between the capacitor plates affect the capacitance, which can be measured with high precision.^[^ ^56^, ^57^, ^58^, ^59^ ^]^ To ensure that the dilatometer exclusively measures the height changes of the stripping electrode without interference from other effects, the setup must maintain constant pressure and isothermal conditions.

**Figure 7:**
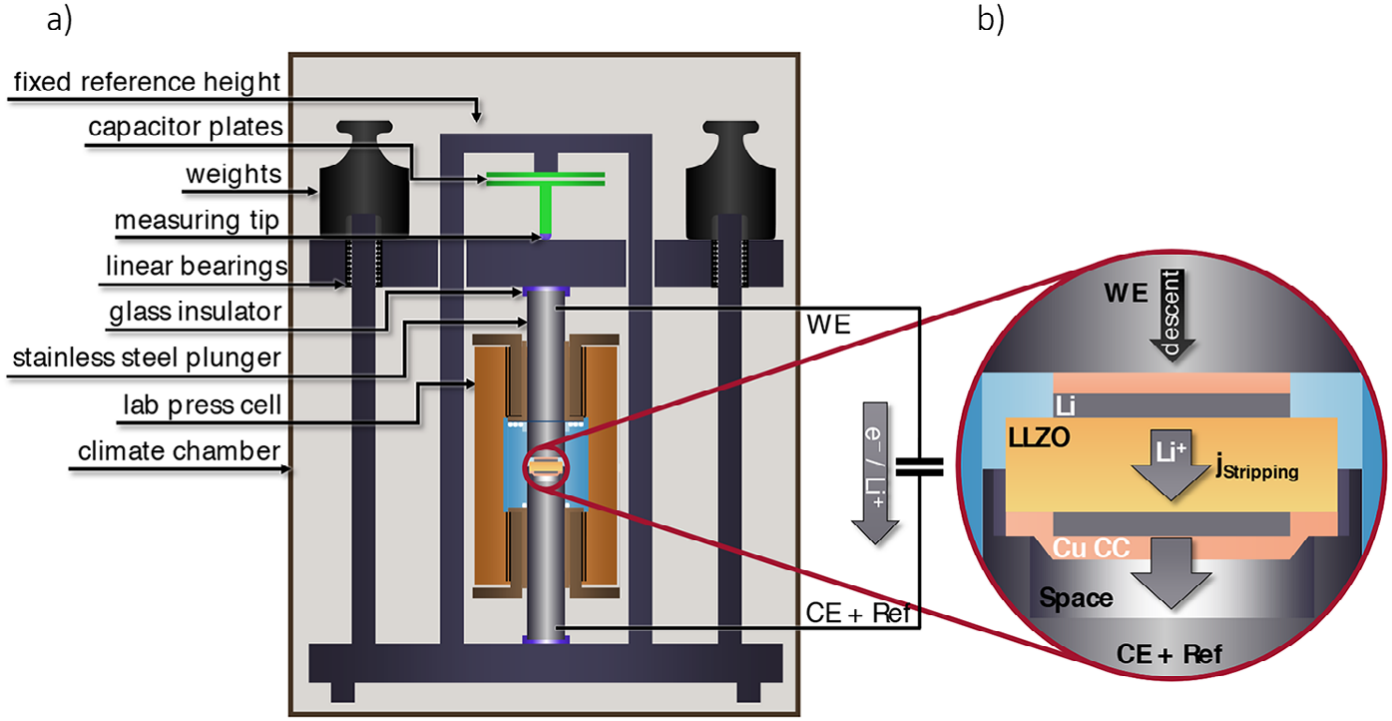
Schematic representation of the dilatometric measurement setup: a) The device maintains constant pressure on a press cell by using weights placed on a plate mounted on linear bearings. The change in height is measured by capacitive dilatometry. b) To exclude the height change of the plating side, it is placed in a recess and therefore mechanically decoupled from the height change measurement (capacitor plates).

A schematic representation of the entire measurement setup was shown in **Figure** **7**a. One capacitor plate was connected to a fixed reference height and the other was connected to the measurement tip. All height changes in the electrochemical cell were mechanically rigidly conveyed to the measuring tip, directly affecting the distance between the capacitor plates (push piston dilatometry). The electrochemical cell was housed within a frictionless press cell. Constant pressure was applied using weights mounted on a plate that moves on linear bearings. Thermal stability (< ± 0.01 K) was ensured by an optimized climate chamber (as discussed later). The plungers of the press cell were connected directly to a potentiostat and electrically insulated from the rest of the measurement setup with borosilicate glass.

To measure only the height change of the stripping electrode, the plating electrode was placed in a recess within the plunger and electrically connected to the plunger and potentiostat via a copper current collector, but not mechanically coupled to the measurement axis (Figure 7b). This means that any height or volume changes associated with lithium deposition on the plating side occur inside the cavity of the recess and were not transferred to the dilatometer, allowing exclusive focus on the effects of the stripping side. The recess has a depth of 2 mm and was filled with argon, providing sufficient space to accommodate deposited lithium without altering the mechanical load path, which remains constant due to the use of fixed weights. Depending on the applied current, the volume of the stripping side decreases, causing a corresponding descent of the plunger, which was mechanically transferred to the measurement tip of the dilatometer (Figure 7a).

A slight tilt between the electrode and the pellet surface cannot be excluded due to surface waviness, minor misalignment during assembly, or geometric deviations from polishing and sintering. However, the resulting measurement error was negligible: Assuming a large tilt of 5°, this results in a vertical deviation of only 0.4% (cos(5°) ≈ 0.996), which was below the resolution limit of the method. Therefore, tilt‐induced deviations were considered insignificant for interpreting the displacement data.

### Measurement Setup—Friction Free Airtight Press Cell

**Figure 8:**
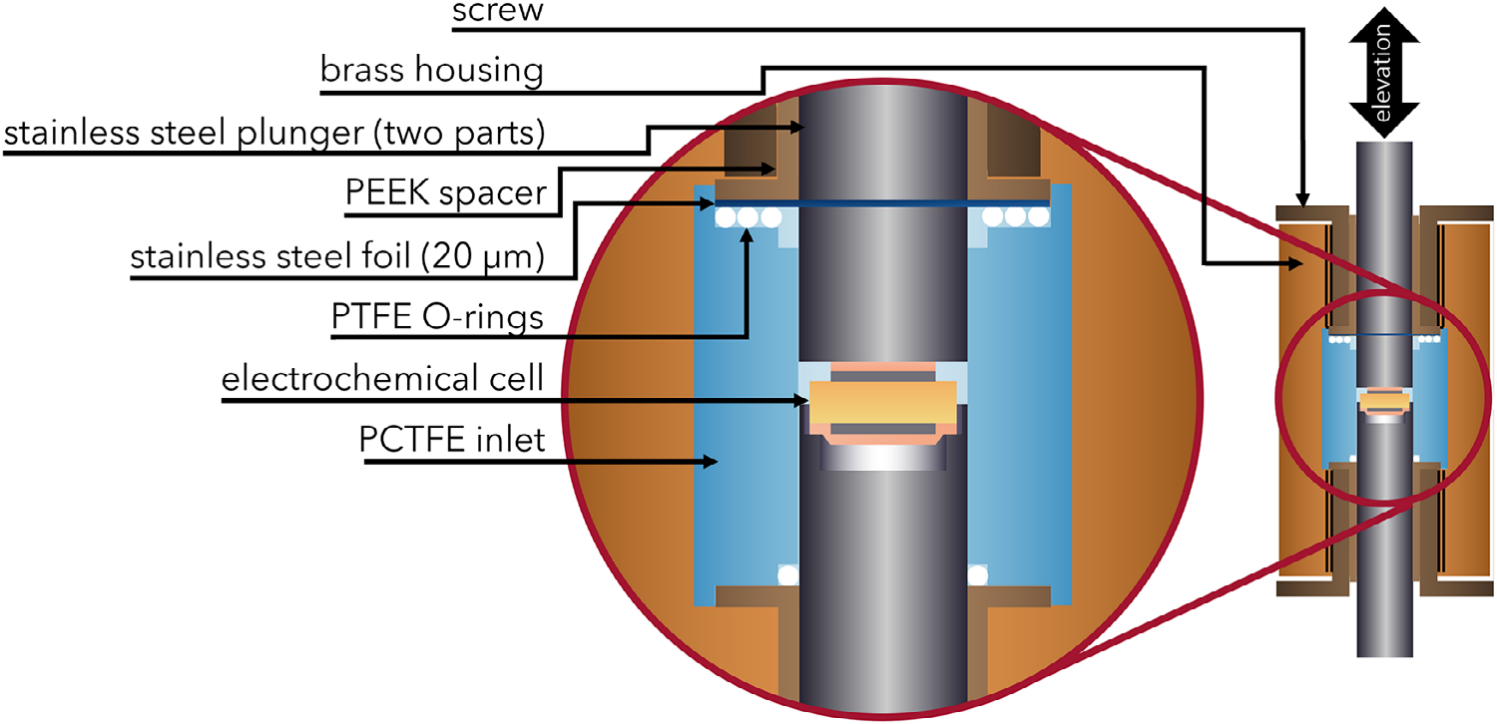
Detailed schematic of the friction free airtight press cell: Friction free movement is provided by a stainless‐steel membrane and sealed airtight with PTFE‐O‐Rings. The electrochemical cell is positioned within a PCTFE inlet, sandwiched between two stainless‐steel plungers. Any height changes on the upper side anode result in a corresponding elevation of the plunger.

To provide high‐accuracy length changes inside the press cells, every height change of the electrochemical cell must be rigidly conveyed to the lower capacitor plate. The change in length must not affect the equilibrium of forces, as this would otherwise result in changes in the length of the elements that were conveying the height change to the capacitor. Frictional force must therefore be avoided, and the plungers cannot be sealed with O‐rings. A stainless‐steel membrane (*1.4301, d*  =  20 µm) was used to seal the cell airtight (**Figure** **8**). The membrane was pressed onto three PTFE O‐rings (⌀18 × 1 mm; ⌀16 × 1 mm, and ⌀14 × 1 mm) using peek spacers. The contact pressure was ensured by tightening the associated screw to 8 Nm, allowing the membrane to move a few 100 µm with negligible restoring force. A short plunger was used to bridge the gap between the membrane and the electrochemical cell. Another plunger was placed above the membrane. As the membrane was also electrically conductive, the working electrode (WE) of the potentiostat can be connected to the plunger above the membrane. The lower plunger was sealed using a PTFE O‐ring (⌀10 × 2 mm*)*. This O‐ring fixes it in place. This cell was based on the cell casing developed by Zhang et.al.^[^ ^60^ ^]^

### Measurement Setup—Height Change Monitoring

The height changes were monitored with the capacitive dilatometer of an EL‐Cell ECD‐4‐nano electrochemical push piston dilatometer. Capacitance changes were measured and converted to height changes by the capaNCDT 6200 controller with a median filter (filter width: 7) applied prior to data transmission, and recorded using the software sensorTOOL 2 (Micro‐Epsilon Messtechnik GmbH & Co. KG). The data was recorded at a frequency of 10.417 Hz. The data were plotted using a custom Python script and smoothed using the Butterworth digital low‐pass filter (scipy.signal.butter). The order of the filter was 1 and the cut‐off frequency was 5·10^−2 ^Hz. The cut‐off frequency was visually optimized and checked. The values were then averaged over 1 s.

### Measurement Setup—Optimized Climate Chamber

The climate chamber KB ECO 240 (BINDER GmbH) was optimized to achieve high‐temperature accuracy by incorporating an additional insulation system within the chamber. For this purpose, an insulation box with a volume of 80 liters was installed inside the climate chamber, housing the measurement device. This additional insulation enabled a temperature stability of 10^−2^ K.

To minimize vibrations and mechanical disturbances, several measures were implemented. The climate chamber utilizes Peltier cooling, which enables vibration‐free cooling. Furthermore, each wheel of the climate chamber was positioned on a damping mat made of cork (22 mm thickness, 100 mm × 100 mm) to suppress vibrations effectively and the climate chamber was located in a windowless room without exterior walls. Access to the room was restricted during measurements to avoid environmental disturbances.

### Measurement Setup—Electrochemical Characterization

Electrochemical impedance and cycling experiments were performed using an SP‐300 potentiostat (BioLogic) and EC‐Lab software (V. 11). The working electrode (WE) was connected to the stripping side, and the counter electrode (CE) and reference (Ref) were connected to the plating side. All galvanostatic electrochemical impedance spectroscopy (GEIS) measurements were carried out in the frequency range between 7 MHz and 1 Hz to keep the system in a quasi‐static state during the measurement. The impedance amplitude was set to 10% of the applied DC current density. The software RelaxIS 3 (RhD Instruments) was used to analyze the impedance data in the frequency domain and to calculate the underlying distribution functions by performing a Distribution of Relaxation Times (DRT) analysis. Low‐quality data were identified and removed using the *Kramers‐Kronig‐Test*. For DRT calculation, only the real part of the impedance data was used. The second derivative of the distribution function *γ*(*τ*) was used in the Tikhonov regularization problem. The regularization parameter λ  = 10^−3^ was used in the calculations.

### Measurement Setup—Temperature Monitoring

To rule out changes in length due to temperature fluctuations, the temperature was continuously monitored before and during the experiment using four Pt‐100 thermocouples. One thermocouple was placed directly on the press cell casing, while two were positioned on the measurement device and the last one outside the inner insulation. Dilatometric measurements were only initiated, when fluctuations were within ± 0.01 K, and only data from measurements where the temperature remained stable within this range throughout the experiment were used. The analog‐to‐digital conversion was performed using a Keithley 2700 digital multimeter with a Keithley 7700 multiplexer. The data were digitally recorded on the measuring PC via serial communication, using a custom Python script.

### Measurement Setup—Active Sample (Piezo Actuator)

The piezo actuator P‐845.20 (Physik Instrumente), with a maximum travel range of 30 µm, was used as the active sample. It was equipped with a strain gauge for precise position determination and was controlled by the E‐709.SRG Digital Piezo Controller. The controller operates in closed‐loop mode, utilizing a built‐in PID servo‐control algorithm to achieve precise positioning.^[^ ^61^ ^]^ Slow movements were implemented using the controller's built‐in wave generator, enabling stepwise displacement in increments as small as 10^−2^ nm, with frequencies adjusted according to the target velocity. The actual position of the actuator was logged digitally by reading the controller's position output.

### Preparation of the Electrochemical Cell—Preparation of Solid Electrolytes

LLZO:Al precursors (Li_2_CO_3_ (>99.0%, Sigma–Aldrich), ZrO_2_ (99.9%, Sigma–Aldrich), La(OH)_3_ (99.9%, Sigma–Aldrich) and Al_2_O_3_ (99.99%, ChemPur) were homogenized using a planetary ball mill for two runs (10 min milling with 20 min pause at 350 rpm for 24 cycles). The precursors were then calcined for 4 h at 1000 °C in MgO crucibles under a 150 sccm O_2_ flow. The calcined precursors were ball‐milled again for 10 min with a 20 min pause at 350 rpm for 40 cycles to obtain a fine powder. This powder was isostatically pressed at 380 MPa into pellets with a diameter of 10 mm and sintered at 1230 °C for 1 h under an O_2_ flow. The sintering procedure was based on the method described in the reference.^[^ ^4^ ^]^ Short sintering times lead to a uniform grain size distribution and small grains.^[^ ^62^ ^]^ All subsequent steps were performed in an argon environment (MBraun, *p*(H_2_O)/*p* < 1.0 ppm , *p*(O_2_)/*p* < 0.1 ppm).

### Preparation of the Electrochemical Cell*—*Cell Assembly

The sintered garnet pellets were polished with grit P1000 SiC‐paper and resistance‐free ideal electrodee Li_id_ were attached on both sides as described in previous publications^[^ ^8^, ^63^ ^]^ using high isostatic pressure (380 MPa for 30 min). Surface degradation layers were removed from a lithium rod with a ceramic knife and then pressed into a thin foil. Afterward, the foil was punched with a ⌀ 6 mm punching tool and pressed with a copper current collector onto the garnet pellet. On the plating side, the copper current collector has a ⌀ 8 mm to allow electrical contact between the plunger and the lithium in the recess. On the stripping side, the copper current collector has a 6 mm diameter, the same as the lithium foil (Figure S7, Supporting Information), to enable precise detection of the exact area with an optical microscope (Leica EMSPIRA‐3). Relative density, roughness, and ionic conductivity of the used LLZO‐Pellets can be found in Table S1 (Supporting Information).

### Preparation of the Electrochemical Cell*—*Microscopic Imaging

Microscopic images of the cell were taken with the Leica EMSPIRA‐3 optical microscope.

### Preparation of the Electrochemical Cell*—*Scanning Electron Microscopy (SEM)

For nanometer‐scale imaging analysis of the pores, the sample was transferred to a Zeiss GeminiSEM 560 HRSEM using the Leica transfer system. The images were taken with the InLens detector at an acceleration voltage of 5 kV.

Data processing and visualization procedures were described in the corresponding sections for height change monitoring and electrochemical characterization.

### Statistical Analysis*—*Dilatometric Data

For the dilatometric data, digital filtering and averaging were applied to suppress high‐frequency noise and improve signal clarity (see Height change monitoring). Data analysis and filtering were conducted using Python (SciPy).

### Statistical Analysis*—*Impedance Data

For impedance data, low‐quality measurements were excluded based on Kramers–Kronig compliance (see Electrochemical Characterization. Data analysis and filtering were conducted using Python (SciPy) and RelaxIS 3 software.

### Declaration of Generative AI and AI‐assisted Technologies in the Writing Process

During the preparation of this work, the authors used ChatGPT (by OpenAI), Copilot (Microsoft), and DeepL Write (DeepL) to improve readability and language. After using this tool, the authors reviewed and edited the content as needed and took full responsibility for the content of the publication.

## Conflict of Interest

The authors declare no conflict of interest.

## Supporting information



Supporting Information

## Data Availability

The data that support the findings of this study are available from the corresponding author upon reasonable request.
