# Supplementary material for: Evolution of Pore Volume During Stripping of Lithium Metal in Solid‐State Batteries Observed with Operando Dilatometry
Source: Small. 2025 Jun 25;21(33):2505053. doi: 10.1002/smll.202505053 (PMC12372434; doi:10.1002/smll.202505053)
Supplement: Supplementary file 1 — Supporting Information [file SMLL-21-2505053-s001.docx]

Supporting Information

**Evolution of Pore Volume during Stripping of Lithium Metal in Solid-State Batteries observed with Operando Dilatometry**

Thomas A. Schall*, Till Fuchs, Janis K. Eckhardt, Tim Klunz, Boris Mogwitz, Joachim Sann, Klaus Peppler, and Jürgen Janek*

E-mail: thomas.schall@phys.chemie.uni-giessen.de, juergen.janek@phys.chemie.uni-giessen.de

((Please insert your Supporting Information text/figures here. Please note: Supporting Display items, should be referred to as Figure S1, Equation S2, etc., in the main text…)

# **I Supplementary Text**

**Calculation of height change for uniform stripping:**

The metal volume corresponding to a given capacity (i.e., charge) is calculated based on Faraday’s law (**Equation S1**).

|  | $V=\frac{Q\cdot M}{F\cdot\rho}$ | (S1) |
| --- | --- | --- |

When assuming a geometry with constant area one can calculate the height change (**Equation S2**; with *q* = *Q* / *A*)

|  | $\Delta h=\frac{Q\cdot M}{A\cdot F\cdot\rho}= \frac{q\cdot M}{F\cdot\rho}$ | (S2) |
| --- | --- | --- |

For an arial capacity of 1 mAh cm^-2^ one can calculate the height change of lithium metal (with 1 mAh cm^−2^ = 3.6 C cm^−2^) as

|  | $\frac{3.6 C \text{c}\text{m}^{-\text{2}}\cdot6.94 \text{g mo}\text{l}^{-\text{1}}}{96485 \text{C mo}\text{l}^{-\text{1}}\cdot0.534 \text{g cm³ }}= 4.85 \mu m$ | (S3) |
| --- | --- | --- |

with a current density of 100 µA cm^−2^ in one minute a charge of 6 mC is transported, which corresponds to a height change of:

|  | $\frac{6\cdot{10}^{-3} C \text{c}\text{m}^{-\text{2}}\cdot6.94 \text{g mo}\text{l}^{-\text{1}}}{96485 \text{C mo}\text{l}^{-\text{1}}\cdot0.534 \text{g cm³ }}= 8.08 nm$ | (S4) |
| --- | --- | --- |

**Calculation of additional descriptors based on the VIR**

The VIR enables the calculation the pore refilling current density ($i_{\text{refill}}$).

|  | $i_{\text{refill}}=i_{\text{stripping}}(1-\text{VIR})$ | (S5) |
| --- | --- | --- |

The pore refilling current density describes the current density with which lithium sites are refilled under the given conditions.

It is assumed that the pore refilling current is pressure-dependent. As the pore area increases and, consequently, the remaining contact area decreases, the local pressure increases. Therefore, the pore refilling pressure is influenced not only by the applied current density (*i*_stripping_), but also by the pore surface. It can be hypothesized that self-diffusion corresponds to the limiting case of the refilling currents for the stripping current approaching zero:

|  | $\lim_{i_{\text{stripping}}\to0} i_{\text{refill}}=i_{\text{self diffusion}}$ | (S6) |
| --- | --- | --- |

**II Supplementary Figures:**


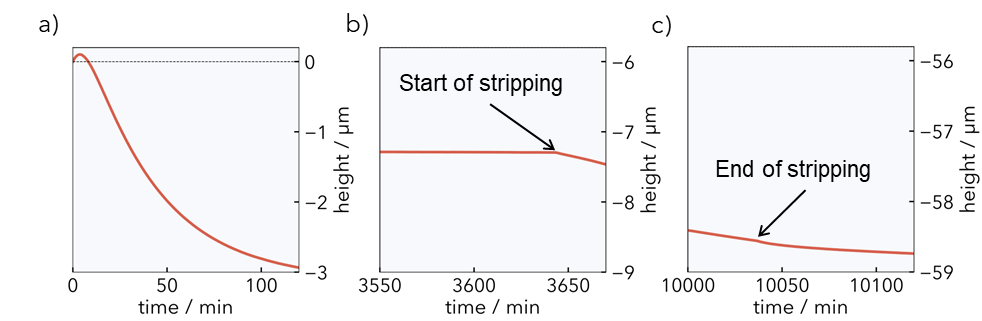


*Figure S1: Relaxation and creep behavior. a) Relaxation and creep immediately after placing the cell in the dilatometer (temperature not yet equilibrated). b) Creep before the onset of stripping. c) Creep after the completion of stripping.*


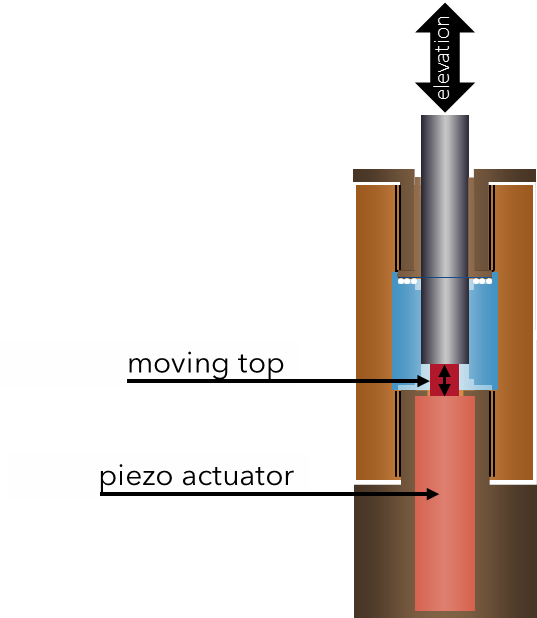


*Figure S2: Schematic of the validation setup: The moving top of a piezo actuator is positioned as an active sample inside the cell.*

*Figure S3: Validation experiments of the measurement setup with piezo actuator (Figure S2).*


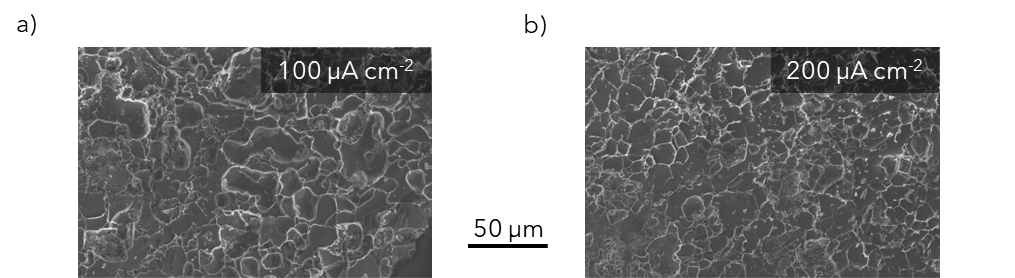


*Figure S4: SEM images of pore formation at an overvoltage of 3 V. The anodes were peeled off from the SE and the images were taken in top view.*

*Figure S5: Total formed pore volume (a) and VIR (b) of the complete data shown in Figure 3. In both measurements, a brief failure in data recording occurred; the missing data were interpolated and are shown as dotted lines.*

*Figure S6: Nyquist-Plots of the GEIS-Measurement with 100 µA cm^–2^ at 0 mAh cm^–2^; 1.4 mAh cm^–2^and 3 mAh cm^–2^ from 1.02 MHz to 1 Hz with corresponding DRT-Plots*


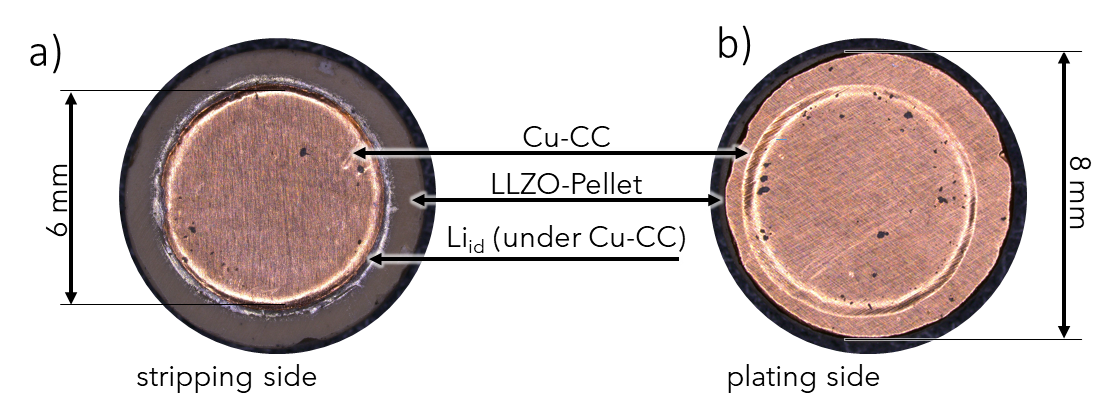


*Figure S7: Exemplary optical microscopy images of an assembled symmetric LLZO half-cell with Li_id_ under a copper current collector. a) stripping side – the copper current collector and the Li_id_ electrode have the same diameter of ⌀ 6 mm. b) plating side – the Li_id_ electrode has a diameter of ⌀ 6 mm and is completely covered by a copper current collector (⌀ 8 mm).*

**III Supplementary Tables:**

Table S 1: Properties of used LLZO-Pellets

| Pellet used in Experiment at | ρ_rel_^a^ [%] | R_a_^b^ [µm] | σ_ion,RT_^c^ [mS·cm⁻¹] |
| --- | --- | --- | --- |
| 100 µA cm^-2^ | 98 | 0.53 | 0.72 |
| 200 µA cm^-2^ | 95 | 0.65 | 0.76 |

1. Relative density calculated from measured pellet weight, height and diameter assuming cylindrical geometry.
2. Average surface roughness R_a_ measured using an optical profiler (Sensofar S Neox) on a circular profile; Gaussian filter with λc = 0.8 mm and λs = 2.5 µm, evaluation length 22.5 mm (ISO 4287).
3. Ionic conductivity determined from the first GEIS measurements at 25 °C of the dilatometric stripping experiment, assuming a 6 mm cylindrical contact area. Slight deviations may occur due to edge effects, as the actual pellet diameter was 8.3 mm. An example spectrum can be found in **Figure S6e** (orange, 0 mAh cm⁻²).
